# Past, present and future of hemophilia: a narrative review

**DOI:** 10.1186/1750-1172-7-24

**Published:** 2012-05-02

**Authors:** Massimo Franchini, Pier Mannuccio Mannucci

**Affiliations:** 1 Immunohematology and Transfusion Center, Department of Pathology and Laboratory Medicine, University Hospital of Parma, Milan, Italy; 2 Scientific Direction, IRCCS Cà Granda Foundation Maggiore Policlinico Hospital, Via Pace, 9, 20122, Milan, Italy

**Keywords:** FVIII, FIX, Plasma-derived factor concentrates, Recombinant factor concentrates, Gene therapy

## Abstract

Over the past forty years the availability of coagulation factor replacement therapy has greatly contributed to the improved care of people with hemophilia. Following the blood-borne viral infections in the late 1970s and early 1980, caused by coagulation factor concentrates manufactured using non-virally inactivated pooled plasma, the need for safer treatment became crucial to the hemophilia community. The introduction of virus inactivated plasma-derived coagulation factors and then of recombinant products has revolutionized the care of these people. These therapeutic weapons have improved their quality of life and that of their families and permitted home treatment, i.e., factor replacement therapy at regular intervals in order to prevent both bleeding and the resultant joint damage (i.e. primary prophylaxis). Accordingly, a near normal lifestyle and life-expectancy have been achieved. The main current problem in hemophilia is the onset of alloantibodies inactivating the infused coagulation factor, even though immune tolerance regimens based on long-term daily injections of large dosages of coagulation factors are able to eradicate inhibitors in approximately two-thirds of affected patients. In addition availability of products that bypass the intrinsic coagulation defects have dramatically improved the management of this complication. The major challenges of current treatment regimens, such the short half life of hemophilia therapeutics with need for frequent intravenous injections, encourage the current efforts to produce coagulation factors with more prolonged bioavailability. Finally, intensive research is devoted to gene transfer therapy, the only way to ultimately obtain cure in hemophilia.

## Introduction

Hemophilia A and B are X chromosome-linked bleeding disorders included among the rare diseases and caused by mutations in the factor VIII (FVIII) and factor IX (FIX) genes [1]. Both factors take part in the intrinsic pathway of blood coagulation and affected individuals have severe, moderate and mild forms of the diseases, defined by factor plasma levels of 1% or less, 2 to 5% and 6 to 40%, respectively. The prevalence of hemophilia A is 1 in 5000 male live births, and that of hemophilia B is 1 in 30,000 [1,2].

Hemophilia was recognized in ancient times. The Talmud, a collection of Jewish rabbinical writings from the second century AD, stated that male babies should not be circumcised provided two brothers had already died owing to excessive bleeding from the procedure. The Arabic physician Albucasis, who lived in the 12^th^ century, described a family with males who died from bleeding after trivial injury [3]. The first modern description of hemophilia is from John Conrad Otto, a physician from Philadelphia, who in 1803 published "An account of an hemorrhagic disposition existing in certain families [4]." He clearly appreciated the cardinal features of hemophilia, i.e., an inherited tendency of males to bleed. However, the first use of the word "hemophilia" appears in an essay written in 1828 by Hopff from the University of Zurich. Hemophilia B was distinguished from the more common hemophilia A in 1952, and was often referred to as "Christmas disease" after the last name of the first child described with this condition [3]. Hemophilia is sometimes referred to as “the royal disease”, because several members of royal families in Europe were affected by this scourge owing to the fact that Victoria, Queen of England from 1837 to 1901, was a hemophilia B carrier [5]. Her eighth son Leopold had hemophilia B, suffered from frequent hemorrhages and died of a brain hemorrhage at the age of 31. Two Queen Victoria’s daughters, Alice and Beatrice, were carriers of hemophilia B and transmitted the disease on to the Spanish, German and Russian royal families [1,6].

The bleeding tendency of hemophilia was originally believed to be due to a fragility of blood vessels. In the 1930s defective platelets were thought to be the most likely cause. Then, in 1937, Patek and Taylor from Harvard found that they could correct the coagulation defect by adding a substance extracted from plasma. This was called *anti-hemophilic globulin*. In 1944, Pavlosky from Buenos Aires, showed that blood from one hemophiliac could correct the coagulation defect of another hemophiliac and vice-versa. He had stumbled upon two patients with a deficiency in different proteins - factor VIII and factor IX [7]. These discoveries permitted an accurate diagnosis, and they also built the basis for the modern therapy of this inherited hemorrhagic disorder. This article will review recent history, actual knowledge and the most important progress and expected improvements in hemophilia care.

## Treatment of hemophilia: the past

In the 1950s and early 1960s, hemophiliacs could be only treated with whole blood or fresh plasma. Unfortunately, there is not enough FVIII or FIX proteins in these blood products to stop severe bleeding. Thus most people with severe hemophilia died in childhood or in early adulthood, hemorrhages after surgery or trauma or in vital organs (especially in the brain) being the most common causes of death [8]. In 1964, the discovery by Judith Pool that the fraction cryoprecipitated from plasma contained large amounts of FVIII represented an enormous step forward in hemophilia care. For the first time enough FVIII could be infused in relatively small volumes to control severe bleeding and major surgery became feasible [9]. However, the modern management of hemophilia truly started in the 1970s, when the increased availability of lyophilized plasma concentrates of coagulation factors and the widespread adoption of home replacement therapy led to the early control of hemorrhages and the reduction of the musculoskeletal damage typical of untreated or poorly treated patients. Primary prophylaxis was successfully pioneered in Sweden and then adopted in other countries, achieving the goal of preventing the majority of bleeding episodes and further reducing the impact of arthropathy [10]. Specialized hemophilia centers became less overwhelmed by the burden of providing emergency treatment, so that they could develop programmes of comprehensive care, with the involvement of such specialists as orthopedic surgeons, physiotherapists, dentists and social workers. Elective surgery, particularly orthopedic operations, helped to correct or minimize the musculoskeletal abnormalities that had developed as a consequence of untreated or inadequately treated bleeding episodes into joints and muscles. In 1977, the discovery of desmopressin, a synthetic drugs that increases plasma levels of FVIII and von Willebrand factor, provided a new, inexpensive and safe treatment for patients with mild hemophilia A (and type 1 von Willebrand disease), who could avoid or substantially reduce the use of plasma-derived products, the corresponding high costs and also the risks of bloodborne infections [1,11].

This optimistic perception of hemophilia changed dramatically in the early 1980s, at a time when 60-70% of people with severe disease became infected with the human immunodeficiency virus (HIV) that had contaminated coagulation factor concentrates. Almost all treated hemophiliacs were also infected with the hepatitis C virus (HCV) (at that time called the non-A, non-B hepatitis virus), transmitted by factor concentrates manufactured from plasma pooled from thousands of donors [12]. As a consequence of the devastating sequelae of the acquired immunodeficiency syndrome (AIDS) and hepatitis epidemics, the need for a safe treatment became crucial for the hemophilia community. The development and implementation of viral inactivation techniques for the production of plasma-derived factor concentrates, as well as the adoption of new methods to screen viruses in blood donation (i.e., NAT testing), greatly improved the safety of plasma-derived products, as shown by the fact that blood-borne transmission of hepatitis viruses or HIV has no longer occurred in the last 25 years [8]. However, the most important advance in this field was represented by the rapid progress in DNA technology (following the cloning in 1982 and 1984 of FVIII and FIX genes), which allowed the industrial production of recombinant FVIII (and subsequently of FIX), culminating with the publication in 1989 of the first report of clinical efficacy of this product in two patients with hemophilia A [13].

Although the safety of plasma-derived factors has dramatically improved in the last 25 years, the fear related to the possible transmission by blood or its derivates of new or unknown pathogens has prompted the hemophilia caregivers of western countries to treat previously untreated hemophilic babies mainly with recombinant products [14]. In parallel, with safety as a priority in mind, also the manufacturing process of recombinant factors evolved during the last few years to further minimize the risk of pathogen transmission, through the improvement of protein purification techniques, the addition of viral inactivation steps and the avoidance of human or animal proteins at any stage of their manufacturing process [15,16].

The availability for replacement therapy of high-quality factor concentrates was important not only for reducing the likelihood of death from hemorrhage but also for the broad implementation of prophylactic treatment regimens in order to prevent bleeding and resultant joint damage, ultimately allowing patients to maintain a near normal lifestyle [17]. This, together with the progress in the management of the blood-borne viral infections through surveillance of patients with chronic hepatitis (especially with respect to hepatocellular carcinoma and liver failure), the availability of newer treatment options such as antiviral treatment against HIV (Highly Active Anti Retroviral Therapy [HAART]) and HCV (combined therapy with α-interferon and ribavirin), greatly contributed to the improved quality of life and reduced morbidity in the hemophilia population [18]. Thus, the last 15 years represent a “new golden era” in hemophilia treatment (after the first “golden era” during the 1970s), with a life expectancy of these patients that progressively approached that of males in the general population, at least in high- and middle-income countries [19]. Table 1 shows the main steps of the advances that occurred and the future goals of hemophilia therapy.

**Table 1:** Past, present and future of hemophilia treatment

**Year**	**Main events**
**1970s: a golden era**	
1970s	Lyophilized factors, home treatment, pioneer prophylaxis programs, comprehensive treatment centers
1977	Desmopressin
**1980s: many shadows, a few lights**	
1982	Factor IX gene cloned, AIDS
1983	Early virucidal methods (dry-heating)
1984	Factor VIII gene cloned, HIV isolated
1985	Anti-HIV testing
1987	Safe virus-inactivated plasma factors
1989	Recombinant FVIII
**1990s: a new golden era**	
1994	Immune tolerance
1996	HAART for HIV
1996	Recombinant FVIIa
1997	Recombinant FIX
**2000s: current hemophilia therapy**	
2000-2006	First gene therapy trials
2002	HCV eradication by IFN-RBV
**The next 10 years**	
2011-2021	More factor concentrate available worldwide Longer-acting recombinant coagulation factors Fusion coagulation factors Cure of hemophilia: gene transfer

## Treatment of hemophilia: the present

In this favorable context, the most challenging complication of therapy has become the development of inhibitory alloantibodies against FVIII or FIX. These inhibitors, that develop in approximately 25-30% of severe hemophilia A patients and in only 3-5% of those with hemophilia B, render replacement therapies ineffective, limit patient access to a safe and effective standard of care and predispose them to an increased risk of morbidity and mortality [20]. The introduction of bypassing agents, such as activated prothrombin complex concentrates (APCC) (Factor Eight Inhibitor Bypassing Activity - FEIBA) and recombinant activated factor VII (rFVIIa, NovoSeven), has dramatically improved the management of acute bleeding in inhibitor patients, allowing home treatment and a substantial amelioration of their quality of life. There has been much debate since the last decade pertaining to the superiority of either product [21]. While the efficacy and safety of FEIBA and rFVIIa appeared to be broadly similar in the context of an inconclusive randomized trial [22], a recent systematic review found that the overall efficacy and bleeding control rates are higher for rFVIIa than for APCC (81–91% and 64–80%, respectively) [23]. Another recent review article, which used a Bayesian meta-regression model to evaluate the outcome of more than 2000 joint bleeds, found that the cumulative rate of control of bleeding at 12, 24 and 36 hours was 66%, 88% and 95% for a standard rFVIIa regimen but was somewhat lower for a standard APCC therapy (39%, 62% and 76%) [24]. By contrast, a Cochrane review published in 2010 on the clinical effectiveness of rFVIIa concentrate in comparison to plasma-derived for the treatment of acute bleeding episodes in people with hemophilia and inhibitors concluded that rFVIIa and APCC have a similar hemostatic effect without increasing the thromboembolic risk [25]. Hence, the issue of superiority of either product is still open, but it is generally accepted that both are quite effective in the control of bleeding episodes in patients with inhibitors.

Despite intensive research in the field of inhibitor development, the mechanism of this complication still remains only partially understood. Patient-related, non-modifiable risk factors as well as environmental modifiable risk factors have been identified [26,27]. Debate on the role of the source of factor used for replacement therapy started after reports on previously untreated patients (PUPs) demonstrated a higher incidence of inhibitors in those treated with recombinant FVIII than in those treated with plasma-derived products [28]. In vitro studies suggested that the presence of von Willebrand factor (VWF) in plasma-derived FVIII concentrates plays a role in decreasing FVIII immunogenicity, via epitope masking and protection of the FVIII molecule from endocytosis by antigen-presenting cells [29-31]. In the attempt to overcome the contrasting and inconclusive literature data [32-34], a systematic review and meta-analysis included as many as 2094 PUPs (1167 on plasma-derived and 927 on recombinant FVIII concentrates) enrolled in 24 prospective and retrospective studies. In this analysis, 14.3% of patients treated with plasma-derived FVIII and 27.4% of patients treated with recombinant FVIII developed inhibitors, and the rate of high-titre inhibitors was higher in patients treated with recombinant FVIII (17.4% versus 9.3%). However, the higher immunogenicity of recombinant FVIII disappeared when a number of possible confounders were included in the analysis [35].

Unfortunately, no randomized clinical trial is currently available to provide definite evidence on whether or not a difference in immunogenicity does indeed exist between plasma-derived and recombinant FVIII. For this reason, the Survey of Inhibitors in Plasma-Product Exposed Toddlers (SIPPET) has recently started (http://www.clinicaltrials.gov, study NCT 01064284; EUDRACT n. 2009-011186-88). SIPPET is an investigator-driven, prospective, randomized, open-label clinical trial comparing inhibitor frequency in PUPs or minimally treated patients first exposed to plasma-derived VWF/FVIII concentrates or recombinant FVIII [36]. At the time of writing, at least one third of the planned number of cases have already been enrolled in SIPPET since the study started. Other large prospective cohort studies of PUPs with severe hemophilia, such as the European PedNet Registry [37] and the French cohort (FranceCoag Network) [38], may also contribute to fulfill these objectives.

Another still controversial issue is that of the comparative efficacy of plasma-derived or recombinant FVIII in antigen-specific immune tolerance induction (ITI), a method meant eradicate inhibitors through the long-term daily treatment of patients with large doses of coagulation factors. ITI is the only proven therapy for inhibitors, but puts enormous challenges on patients and the community in terms of venous access and economic burden (up to 1 million Euros), so that it is prohibitive for many countries. After several in vitro studies showed a decrease in inhibitor activity against FVIII complexed with VWF (VWF/FVIII) compared with that against recombinant FVIII [39,40], a number of clinical studies have explored the role of FVIII source in ITI, and some clinical experience in Europe and the USA suggest that plasma-derived FVIII products rich in VWF may increase the likelihood of successful ITI [41]. In this context, the results of two large ongoing prospective randomized trials, the Rescue Immune Tolerance Study (RESIST)-naive (ITI-naive patients with poor prognostic factors are randomly assigned to VWF/FVIII or recombinant FVIII at a dose of 200 IU/kg/day) and RESIST-experienced (patients who have previously failed ITI with monoclonal or recombinant FVIII undergo ITI using VWF/FVIII containing plasma-derived concentrates at a daily dose of 200 IU/kg), will perhaps help us to elucidate the role of FVIII source in ITI [42].

With the widespread adoption of primary prophylaxis programs and the implementation of ITI regimens requiring intensive and regular infusion of factor concentrates, the route of administration of replacement therapy has become a crucial issue. Although peripheral venipuncture is the first choice, central venous access devices (CVADs) are often necessary in very young children with poor venous access. Totally implantable catheters (ports) should be preferred over external CVADs because the risk of complications, especially infections and thrombosis, is smaller [43]. Transient arteriovenous fistulae are also a promising option for children of one year of age and older (in particular for those who have experienced repeated CVAD failure) [44], with positive results reported in two studies conducted in Italy and USA [45-47]. However, this approach requires highly experienced surgeons, a continued follow-up and should be performed only in specialized centers.

While primary prophylaxis remains the gold standard for preserving joint function in babies with severe hemophilia [48], there is actually a debate regarding the effects on joint status of secondary prophylaxis compared with on demand therapy in older children, adolescents and adults [49]. In this context, considering also that all published data are derived from small retrospective studies, the results of two prospective studies, the SPINART (Trial to Evaluate the Effect of Secondary Prophylaxis With Recombinant FVIII Therapy in Severe Hemophilia A Adult Subjects Compared to That of Episodic Treatment) and the POTTER (Prophylaxis vs. On-demand Therapy Through Economic Report) trials, are awaited by the scientific community [50].

While reaching more advanced age, persons with hemophilia are developing medical and surgical conditions, such as cardiovascular diseases and cancers, that were previously uncommon [17,18,51-53]. These diseases represent novel causes of morbidity and mortality, whose management represents a challenge for hemophilia caregivers [54,55]. Their optimal management requires a close cooperation among physicians from different specialties such as hematology, oncology, cardiology, nephrology, surgery and internal medicine. Unfortunately, only few data are available on health care issues of elderly patients with hemophilia so that treatment recommendations have a low grade of evidence, being mostly based on the personal experience of panels of experts [56,57]. Well-designed clinical trials are awaited to optimize the management of comorbidities, especially cardiovascular disorders and cancer, in elderly hemophiliacs.

In the last decade the ultimate goal of the investigators has been the search of the definitive cure of hemophilia, i.e. the correction through gene transfer of the underlying DNA defect [58]. Following the excellent results obtained in animal models of hemophilia [59,60], at the beginning of the third millennium a few phase 1/2 studies of somatic cell gene therapy conducted in patients with FVIII or FIX deficiency appeared to support the great expectations. In the majority of them, using in vivo or ex vivo approaches to transfer the normal gene, measurable levels of FVIII or FIX could be attained in plasma [61-64]. Although these early promising results generated considerable optimism, further studies did not fulfill the initial expectations and dampened excessive enthusiasm. The problems of transient and therapeutically insufficient factor levels achieved in plasma through the transferred normal gene and, most importantly, severe side-effects such as reactions of the host immune system to viral vectors and the risk of insertional mutagenesis, decreased enthusiasm for further studies in humans [65]. On the other hand, because studies in animal models have become more and more successful, two clinical studies have recently started in hemophilia B patients based upon the use of adeno-associated virus (AAV) vectors for gene transfer [8]. The genetic induction of FVIII and FIX expression in megakaryocytes/platelets or endothelial cells [66,67] is also being explored, an approach that has the potential to restore local hemostasis at sites of vascular injury and thus to be effective also in patients with inhibitors.

## The treatment of hemophilia: the future

In the last two decades the forementioned advances on the efficacy and safety of the treatment of hemophiliacs have been obtained and implemented quasi exclusively in western countries. Thus, the first goal for the next future is to obtain wider treatment availability. There are a number of emerging and densely populated countries, such as India and China, where the level of hemophilia care is far from being satisfactory. For these countries, which are rapidly developing a high level of technological competence, it is probably more appropriate to foster DNA technology with the goal to produce recombinant factors and develop gene transfer rather than programmes based on plasma fractionation. On the other hand, the industrial production of plasma-derived factors should continue and expand, to meet the increasing needs and demands of those countries (specially in South America and Eastern Europe) that are rapidly improving their programmes of health care delivery to persons with hemophilia and that cannot afford the higher cost of recombinant factors. Although the extension of programmes of hemophilia care to developing countries is the main goal for the immediate future, there are also a number of objectives for high-income countries. First of all, to maintain the current excellent levels of treatment that risks to be jeopardized owing to the global economic crisis. It must be emphasized that the costs of hemophilia are truly a tiny part of the whole budget for health care in any country, and that cost effectiveness of hemophilia care is well proven. In addition, richer countries should support the needs of factor replacement therapy of low-income countries of Africa, where an adequate production of plasma-derived or recombinant factor concentrates cannot be foreseen for the next future.

From a research point of view, which advances in the treatment are likely to be realized in the next ten years? The most likely progress in this field is the availability of FVIII and FIX molecules with longer half-lives. This would be a significant step forward, considering that in countries that can afford primary prophylaxis the main obstacles to its widespread adoption are problems related to venous access. Several pharmaceutical companies are currently developing factors with longer half-life to obviate frequent administration, and/or reduced antigenicity/immunogenicity to minimize inhibitor development [68-70]. The main strategies being applied to FVIII include modifications of the molecule, such as the addition of polyethylene glycol (PEG) polymers or polysialic acids, and alternative formulation with PEG-modified liposomes (PEGLip) [71]. The last approach was used to produce BAY79-4980 (Bayer Schering Pharma AG, Germany), which prolonged the bleeding free period in the frame of phase I studies [72]. However, in January 2010 a phase II trial (the LIPLONG study, comparing the PEGLip formulation with standard Kogenate FS) carried out in 62 centers in 14 different countries was prematurely discontinued because the study was unable to achieve the predetermined efficacy endpoint (noninferiority). Research on longer-acting PEGylated recombinant factor VIIa (rFVIIa) showed the ability of this new product to activate factor X on tissue factor expressing cells [73]. In addition, a recently published phase I/II study comparing the safety and efficacy of the PEGLip rFVIIa (Novo Nordisk, Bagsvaerd, Denmark) with the standard rFVIIa in severe hemophilia A patients with inhibitors found that the PEGylated formulation yielded an improved hemostatic efficacy without increasing the risk of thrombosis [74]. Improved pharmacokinetics properties of coagulation factors have also been obtained through molecule modification by genetic fusion with albumin and the IgG Fc moiety [75,76]. Finally porcine FVIII, originally derived from animal plasma and endowed with the property of low cross-reactivity to human FVIII inhibitors, was recently produced by recombinant DNA technology as a B-domainless molecule **(OBI-1)** and tested in a phase II trial in hemophilia A patients with inhibitors [77]. This preparation was effective in controlling bleeding and was well tolerated [77]. However, further studies are needed to evaluate the immunogenity of OBI-1.

## Conclusions

In the past three decades, hemophilia has moved from the status of a neglected and often fatal hereditary hemorrhagic disorder to that of a defined group of well-characterized molecular entities. There is little doubt at the moment that, among the most prevalent monogenic disorders (cystic fibrosis, thalassemia, muscular dystrophy), hemophilia enjoys the most efficacious and safe treatment. Indeed, after the dramatic events of widespread blood-borne virus transmission in the 1970s–1980s, there has been a strong drive towards a continuous improvement in the efficacy and safety of replacement therapy and towards the cure of the disease through gene therapy. To maintain this high level of health care and research two elements are essential. First, there is a need of international collaboration in clinical research on hemophilia. Indeed, very few of the aforementioned cogent unsolved questions can be tackled by studies done in single, albeit large, hemophilia centers. The adequacy of the sample size is essential also for a rare disease such hemophilia and this goal can be achieved only through collaborative multicenter studies. Secondly, it is necessary to maintain a high interest and expertise in the field of hemophilia, especially among newer generations of physicians, who appear to be attracted by the more appealing thrombotic side of hemostasis [78].

## Competing interests

PMM has received speaker fees from Baxter, Bayer, Biotest, Grifols, Kedrion, Novo Nordisk, Pfizer and consultantship fees from Bayer. MF declares no conflict of interest.

## Authors’ contributions

MF wrote the first draft of the manuscript after detailed planning with PMM, who revised substantially all the drafts. Both authors read and approved the final manuscript.
